# Nursing students’ relationships among meaning in life, well-being, and positive beliefs: A cross-sectional survey study

**DOI:** 10.1097/MD.0000000000012914

**Published:** 2018-10-19

**Authors:** Fu-Ju Tsai, Cheng-Yu Chen, Gwo-Liang Yeh, Yih-Jin Hu, Chie-Chien Tseng, Si-Chi Chen

**Affiliations:** a Department of Health Promotion and Health Education, National Taiwan Normal University, Taipei; b Department of Nursing, Fooyin University School of Nursing, Kaohsiung; c Department of Education, National Taipei University of Education, Taipei, Taiwan.

**Keywords:** health promotion, meaning in life, nursing students, positive beliefs, well-being

## Abstract

Nursing educators have a core responsibility to develop nursing students’ health promotion. The purpose of this study was to explore nursing students regarding their relationships among meaning in life, well-being, and positive beliefs. A cross-sectional survey design was adopted in this study. Purposive sampling was used. A total of 219 nursing students participated in the study. Quantitative analysis was utilized for the data analyses. The study found that nursing students had the following mean scores on the meaning in life, 4.33 (86.60%) standard deviation (SD) 0.79; well-being, 4.23 (84.60%) SD 0.78; and positive beliefs, 4.32 (86.40%) SD 0.75. The nursing students’ characteristics, meaning in life, and well-being explained 79% of the variance in positive beliefs (adjusted *R*^*2*^ = 0.79, *F* = 114.57, *P* < .001). Nursing educators can promote meaning in life and well-being to nursing students as an effective way to increase their positive beliefs.

## 1 Introduction

In the 21st century, many people suffer from physical, psychological, spiritual, and social health problems that are related to numerous diseases, including cancer, stroke, cardiovascular disease, mental problems, stress,^[1]^ depression,^[2]^ and others. Positive beliefs, however, can lead to effective management of health problems, which can result in an overall healthy life. Extant literature has demonstrated positive beliefs^[3]^ to be related to meaning in life^[4]^ and well-being, that lead to a high quality of life.^[5]^ A sense of meaning in life may also promote positive beliefs and improve quality of life for better health promotion.^[6]^ A pleasant life, positive emotions, positive attitudes, positive beliefs, and meaning in life also develop well-being and life satisfaction.^[7,8]^ In addition, positive beliefs constitute a significant predictor of well-being, meaning in life,^[9,10]^ creative daily life,^[11]^ and efficacious management of health problems.^[1]^ Therefore, positive beliefs have been shown to facilitate successful management of peoples’ physical, psychological, spiritual, and social health problems^[2]^ to promote the meaning in life and well-being in daily life.

Nursing students should possess healthy attitudes, positive beliefs, and nursing abilities to optimally take care of patients.^[12]^ Consequently, nursing educators worldwide need to encourage nursing students to have a positive lifestyle, positive behaviors, and positive beliefs so that they can be of maximum service in their work.^[13]^ A healthy workplace is also closely related to nursing students’ physical, psychological, spiritual, and social health promotion.^[14]^ A healthy workplace promotes a feeling of joy, a perception of well-being, and an understanding of the meaning in life. Moreover, positive beliefs may create habits of positive thinking that involve finding meaning in life in daily life,^[15]^ so that nursing students can provide better holistic care to their patients.^[14]^ Using meaning in life^[4]^ and positive beliefs can also assist patients in changing their negative moods, ameliorating their psychological problems,^[16,17]^ and increasing mental health and quality of life.^[18]^ Overall, empowering positive beliefs may increase patients’ creativity, cognition, and growth to have a better quality of life, reduce mental illness, and increase health promotion.^[19]^

Psychological well-being may be acquired from daily life experiences to improve quality of life.^[20]^ Nursing students should offer holistic health practices to patients for increasing their psychological well-being and promoting quality of life.^[21]^ Each person may have his or her own sense of well-being, ideas, and feelings in daily life. Well-being can enhance personal growth, self-acceptance, self-actualization, an independent personality, good relationships, a purpose in life, and vitality.^[8]^ Furthermore, well-being can be divided into subjective and psychological well-being.^[22]^ Subjective well-being is an essential component for training nursing students. Promoting peer care and resilience can improve nursing students’ subjective well-being.^[23]^ Training nursing students in resilience and coping strategies^[24]^ helps them to manage stress and increases psychological well-being.^[25]^ Improving subjective and psychological well-being^[22]^ entails promoting nursing students’ physical, psychological, spiritual, and social health promotion. Because nursing students have a duty to take care of patients and solve many of their problems, they should lead a healthy lifestyle and have a high quality of life. Therefore, subjective and psychological well-being is closely related to the relationships between nursing students’ physical, psychological, spiritual, and social health promotion.

After completing their nursing programs, nursing students should devote themselves to promoting the quality of life of their patients. Nursing students also often face the life and death problems of their patients, and can contribute substantially to relieving patients’ problems through their skills in physical, psychological, spiritual, and social health promotion. Moreover, nursing students should be equipped with the specific professional competences to take care of a broad range of patient types. For these reasons, nursing educators are responsible to equip nursing students with respect for the influence of meaning in life, well-being, and positive beliefs on physical, psychological, spiritual, and social health promotion. Therefore, nursing educators need to understand nursing students’ relationships among meaning in life, well-being, and positive beliefs to improve the quality of life of their patients.

### 1.1 Purpose

The purpose of this study was to explore nursing students’ relationships among meaning in life, well-being, and positive beliefs.

## 2 Methods

### 2.1 Design

A cross-sectional study with a quantitative approach was adopted.

### 2.2 Framework

**Figure 1:**
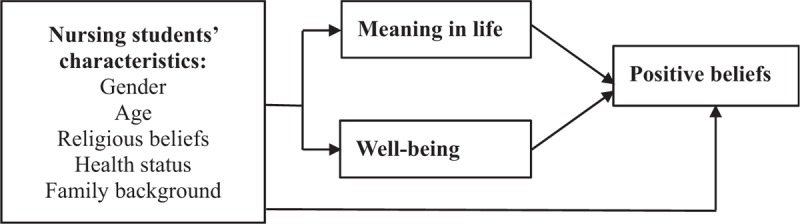
The framework of this study.

The framework of this study aimed to investigate the characteristics of nursing students in terms of gender, age, religious beliefs, health status, and family background, in relation to the meaning in life, well-being, and positive beliefs (Fig. 1).

### 2.3 Participants

Purposive sampling was used in this study. The researcher selected all 219 3rd-year nursing students enrolled in a 5-year nursing program from the curriculum of health promotion at a technology university.

### 2.4 Ethical considerations

The protocol of this study was approved by the Institutional Review Board of Yuan's General Hospital (IRB no: 20171130B) in Taiwan.

### 2.5 Instruments

The study instruments were the Life Attitude Profile by Ho^[26]^ and the Positive Coping, Spirituality, and Well-Being Scale by Lin and Yu.^[27]^ A 56-item questionnaire was used to measure meaning in life, well-being, and positive beliefs. The questionnaire included nursing students’ gender, age, religious beliefs, health status, family background, meaning in life (1–25 items), well-being (1–20 items), and positive beliefs (1–11 items). A 5-point Likert-type scale, ranging from completely disagree (1) to completely agree (5), was employed for this research. The content validity index of the study questionnaire was 0.95, as established by seven expert scholars. The reliabilities of the study on the 3-part measure (n = 61) were as follows: meaning in life had a Cronbach α of 0.96; well-being had a Cronbach α of 0.95; and positive beliefs had a Cronbach α of 0.93.

### 2.6 Data collection

The researcher administered the survey questionnaires to 254 nursing students and explained that these questionnaires aimed to explore nursing students’ relationships among meaning in life, well-being, and positive beliefs. All of the nursing students could decide to completely or partially fill out the survey questionnaires. The nursing students self-responded to the 56 questions regarding meaning in life, well-being, and positive beliefs. Finally, 86.22% of the questionnaires were completed, and the loss of questionnaires totaled 13.78%. The researcher collected all completed 219 questionnaires from January 8, 2018 to January 19, 2018.

### 2.7 Data analysis

The SPSS 23.0 statistical package was utilized to analyze all of the data for this study. Data analysis included percentages, frequencies, means, standard deviations (SDs), 1-way analysis of variance by rank, 1-way analysis of variance, Spearman rho correlation, and regression analysis.

## 3 Results

The study participants comprised 219 nursing students in a curriculum of health promotion. The results of the study presented the gender, age, religious beliefs, health status, family background, meaning in life, well-being, and positive beliefs of the nursing students.

Regarding gender distribution, the 219 nursing students consisted of 21 (9.60%) males and 198 (90.40%) females. In terms of age distribution, the total group included 141 (64.40%) 17-year olds and 78 (35.60%) 18-year olds. Regarding religious beliefs, 219 nursing students included 94 (42.90%) with no religious beliefs and 125 (57.10%) with religious beliefs. Regarding health status, the total group included 117 (53.40%) with normal health status and 102 (46.60%) with very good health status. In terms of family background, 219 nursing students included 171 (78.10%) who were raised by both parents and 48 (21.90%) who were raised by others.

### 3.1 Spearman rho correlation analysis on meaning in life, well-being, and positive beliefs

Spearman rho correlation analysis indicated that the 219 nursing students on meaning in life were positively correlated with well-being, *r* = 0.859 (*P* < .01) and positive beliefs, *r* = 0.887 (*P* < .01) (Table 1). Furthermore, for the 219 nursing students, well-being was positively correlated with meaning in life, *r* = 0.859 (*P* < .01) and positive beliefs, *r* = 0.832 (*P* < .01) (Table 1). In addition, all of the nursing students had positive beliefs that were positively correlated with meaning in life, *r* = 0.887 (*P* < .01) and well-being, *r* = 0.832 (*P* < .01) (Table 1).

**Table 1:**
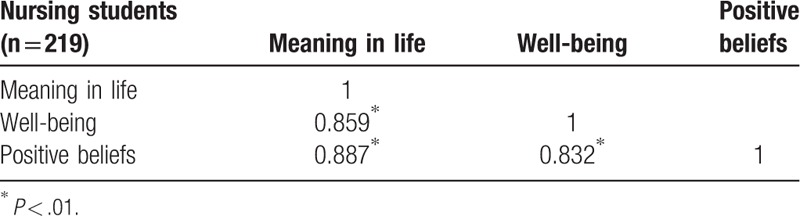
Spearman rho correlation analysis on meaning in life, well-being, and positive beliefs.

### 3.2 Nursing students’ mean scores on meaning in life, well-being, and positive beliefs

The 219 nursing students had an adequate sense of meaning in life, with a mean score of 4.33 (86.60%) SD 0.79; well-being, with a mean score of 4.23 (84.60%) SD 0.78; and positive beliefs, with a mean score of 4.32 (86.40%) SD 0.75 (Table 2).

**Table 2:**
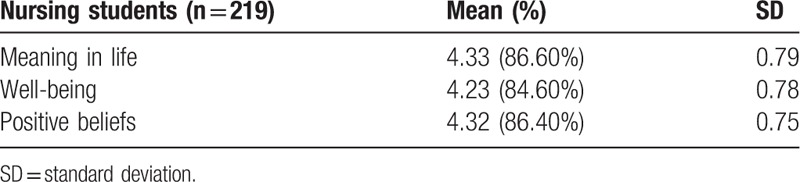
Nursing students’ mean scores on meaning in life, well-being, and positive beliefs.

### 3.3 Nursing students’ characteristics and meaning in life

Nursing students’ characteristics on health status indicated significant relationships with meaning in life. Nursing students had a very good health status, with mean scores of 4.60 that exhibited a close relationship with meaning in life (*P* < .01) (Table 3).

**Table 3:**
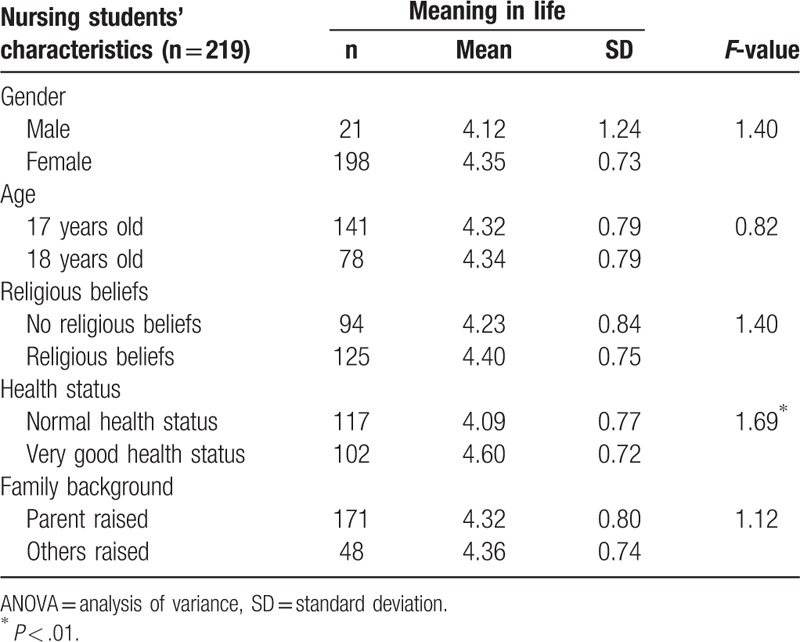
1-way ANOVA of nursing students’ characteristics and meaning in life.

### 3.4 Nursing students’ characteristics and well-being

Nursing students’ characteristics on health status constituted a strong indicator of the relationship with well-being. Nursing students had a very good health status, with a mean score of 4.50 that showed a close relationship with well-being (*P* < .001) (Table 4).

**Table 4:**
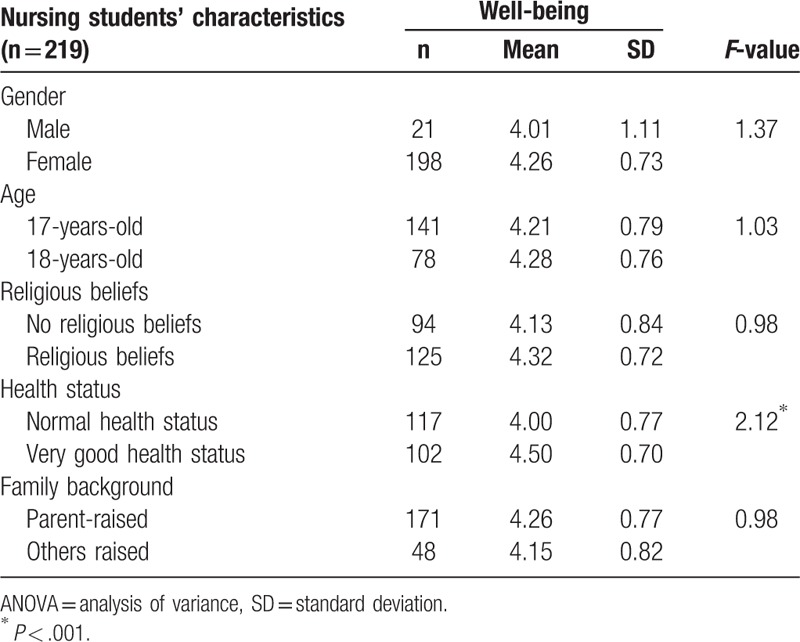
1-way ANOVA of nursing students’ characteristics and well-being.

### 3.5 Nursing students’ characteristics and positive beliefs

Nursing students’ characteristic on health status was strong indicator of the relationship with positive beliefs. Nursing students had a very good health status, with a mean score of 4.61 that exhibited a close relationship with positive beliefs (*P* < .001) (Table 5).

**Table 5:**
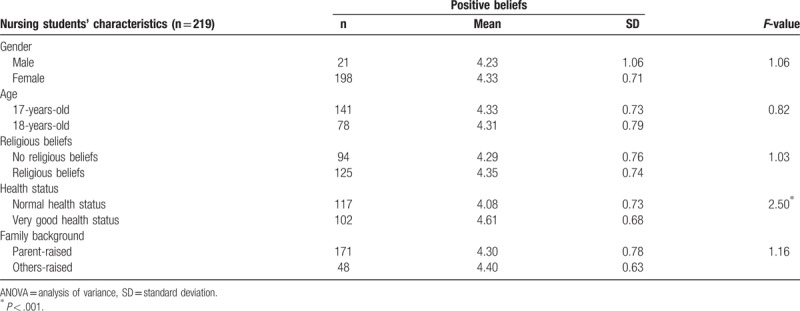
1-way ANOVA of nursing students’ characteristics and positive beliefs.

### 3.6 Nursing students’ characteristics, meaning in life, and well-being to predict positive beliefs

Nursing students’ characteristics, meaning in life, and well-being explained 79% of the variance in positive beliefs (adjusted *R*^2^ = 0.79, *F* = 114.57, *P* < .001) (Table 6). Table 6 presents the data on family background (*B* = −0.12, *t* = −2.10, *P* < .05), meaning in life (*B* = 0.58, *t* = 10.60, *P* < .001), and well-being (*B* = 0.29, *t* = 5.15, *P* < .001). The results revealed that meaning in life and well-being had the strongest impact on nursing students’ positive beliefs (Table 6). Moreover, family background of nursing students had the second-highest impact on their positive beliefs (Table 6).

**Table 6:**
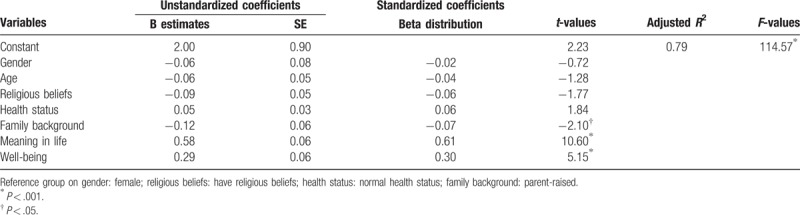
Regression analysis on nursing students’ characteristics, meaning in life, and well-being to predict positive beliefs.

## 4 Discussion

After much suffering, people can become more aware of the experience of the event that caused the suffering, and change their lives, receive empathy, accept others, find a meaning in life, create well-being, etc.^[28]^ People typically regard the meaning in life as constituting certain goals or purposes,^[29]^ and seek relationships with others, personal growth, and religion to improve their understanding of the meaning in life and well-being.^[30]^ In this research, meaning in life constitutes an important factor that is positively associated with peoples’ optimism, self-esteem, self-actualization, and positive effect on increasing psychological health and decreasing numerous fears in daily life.^[31]^ Psychological well-being comprises autonomy, personal growth, purpose in life, self-acceptance, and positive relationships with others. Indeed, numerous previous studies have demonstrated positive relationships among meaning in life, well-being, and positive beliefs.^[32]^

Much extant research reports clear relationships between meaning in life, well-being, and positive beliefs. The results of this research were consistent with the findings of these other articles. According to the study results, the average scores of nursing students are as follows: meaning in life 4.33, well-being 4.23, and positive beliefs 4.32. Consequently, nursing educators should be aware of the relationships existing between meaning in life, well-being, and positive beliefs to promote their understanding to nursing students in the future. In today's nursing education, nursing educators must motivate nursing students to possess their own meaning in life and well-being to generate their own positive beliefs, in order to promote physical, psychological, spiritual and social health, and take care of people effectively in clinical communities worldwide.

People's meaning in life can promote and encourage a sense of beauty, joy, happiness, and well-being in daily life.^[33]^ People regard the meaning in life to be the same as life goals^[29]^ and seek relationships with religion to increase their meaning in life and well-being.^[30]^ A person's sense of the meaning in life may also promote psychological health and healthy behaviors.^[34]^ In addition, well-being may impact positive beliefs in daily life,^[35]^ and meaning in life may promote well-being and positive beliefs.^[15]^ Therefore, positive relationships are shown to exist among meaning in life, well-being, and positive beliefs.

In the results of this study, nursing students had a sense of meaning in life that was positively associated with well-being; conversely, nursing students had well-being that was positively correlated with meaning in life. Moreover, nursing students’ characteristics, meaning in life, and well-being were combined to explain 79% of the variance in positive beliefs. Nursing students’ sense of meaning in life and well-being had the highest impact on positive beliefs, with family background having the second-highest impact. Based on these findings, nursing educators should recognize and carefully teach nursing students that meaning in life and well-being impact their positive beliefs and improve physical, psychological, spiritual, and social health promotion, as well as substantially impact their ability to take care of the many patients in the world.

### 4.1 Limitations

The limitations of this study is that the sample was limited to 219 nursing students in a 5-year nursing program. All participants were in their 3rd year nursing promotion and attended in the health promotion curriculum. This may have limited the data that were collected. In addition, participants were limited to nursing students in the department of nursing at a university in Kaohsiung City, Taiwan.

## 5 Conclusion

The study found that nursing students had a mean score on meaning in life of 4.33, well-being of 4.23, and positive beliefs of 4.32. All nursing students had a meaning in life that was positively correlated with well-being, *r* = 0.859; conversely, all nursing students had a well-being that was positively correlated with meaning in life, *r* = 0.859. In addition, nursing students’ characteristics, meaning in life, and well-being explained 79% of the variance in positive beliefs. Moreover, nursing students’ meaning in life and well-being had the greatest impact on their positive beliefs, and family background had the second-highest impact on their positive beliefs. Furthermore, nursing students’ sense of meaning in life and well-being may impact their positive beliefs. Therefore, nursing educators can promote meaning in life and well-being to nursing students as an effective way to increase their positive beliefs for optimal physical, psychological, spiritual, and social health promotion in patient care.

## Author contributions

**Conceptualization:** Fu-Ju Tsai, Yih-Jin Hu.

**Data curation:** Fu-Ju Tsai, Yih-Jin Hu.

**Formal analysis:** Fu-Ju Tsai, Cheng-Yu Chen.

**Funding acquisition:** Fu-Ju Tsai.

**Investigation:** Fu-Ju Tsai.

**Methodology:** Fu-Ju Tsai, Cheng-Yu Chen, Yih-Jin Hu.

**Project administration:** Fu-Ju Tsai.

**Resources:** Fu-Ju Tsai.

**Software:** Fu-Ju Tsai.

**Supervision:** Gwo-Liang Yeh, Yih-Jin Hu, Chie-Chien Tseng, Si-Chi Chen.

**Validation:** Gwo-Liang Yeh, Yih-Jin Hu, Chie-Chien Tseng, Si-Chi Chen.

**Visualization:** Gwo-Liang Yeh, Yih-Jin Hu.

**Writing – original draft:** Fu-Ju Tsai.

**Writing – review & editing:** Yih-Jin Hu.
